# Role of Bladder Cancer Metabolic Reprogramming in the Effectiveness of Immunotherapy

**DOI:** 10.3390/cancers13020288

**Published:** 2021-01-14

**Authors:** Mathijs P. Scholtes, Florus C. de Jong, Tahlita C. M. Zuiverloon, Dan Theodorescu

**Affiliations:** 1 Department of Urology, Erasmus MC Cancer Institute, Erasmus University Medical Center Rotterdam, 3015 GD Rotterdam, The Netherlands; m.scholtes@erasmusmc.nl (M.P.S.); f.c.dejong@erasmusmc.nl (F.C.d.J.); t.zuiverloon@erasmusmc.nl (T.C.M.Z.); 2 Cedars-Sinai Samuel Oschin Comprehensive Cancer Institute, Los Angeles, CA 90048, USA; 3 Cedars-Sinai Medical Center, Department of Surgery (Urology), Los Angeles, CA 90048, USA; 4 Cedars-Sinai Medical Center, Department of Pathology and Laboratory Medicine, Los Angeles, CA 90048, USA

**Keywords:** bladder, urothelial cancer, metabolic reprogramming, immunotherapy

## Abstract

**Simple Summary:**

Up to 90% of bladder cancers originate from the cells that line the interior of the bladder and are called urothelial carcinomas (UC). Faster growth of UC leads to a higher demand for nutrients and energy than non-malignant cells. UC compensates for this high demand for energy and building blocks by upregulation of different metabolic and bioenergetic pathways, in a process which is known as metabolic reprogramming (MR). However, this MR creates an environment within the tumor that alters immune cells, which in turn reduces the effectiveness of anticancer treatments such as immunotherapy. Here, we review UC MR and its impact on immune cells in UC in order to explore research opportunities that may improve immunotherapy. We discuss the current understanding of UC MR in animal models and summarize clinical trials that are investigating metabolism as a target to enhance immunotherapy in UC patients.

**Abstract:**

Metabolic reprogramming (MR) is an upregulation of biosynthetic and bioenergetic pathways to satisfy increased energy and metabolic building block demands of tumors. This includes glycolytic activity, which deprives the tumor microenvironment (TME) of nutrients while increasing extracellular lactic acid. This inhibits cytotoxic immune activity either via direct metabolic competition between cancer cells and cytotoxic host cells or by the production of immune-suppressive metabolites such as lactate or kynurenine. Since immunotherapy is a major treatment option in patients with metastatic urothelial carcinoma (UC), MR may have profound implications for the success of such therapy. Here, we review how MR impacts host immune response to UC and the impact on immunotherapy response (including checkpoint inhibitors, adaptive T cell therapy, T cell activation, antigen presentation, and changes in the tumor microenvironment). Articles were identified by literature searches on the keywords or references to “UC” and “MR”. We found several promising therapeutic approaches emerging from preclinical models that can circumvent suppressive MR effects on the immune system. A select summary of active clinical trials is provided with examples of possible options to enhance the effectiveness of immunotherapy. In conclusion, the literature suggests manipulating the MR is feasible and may improve immunotherapy effectiveness in UC.

## 1. Introduction

Worldwide, there were approximately 550,000 new cases and 200,000 deaths from bladder cancer (BC) in 2018 [1]. Up to 90% of BC cases originate from the luminal urothelial lining of the bladder and produce urothelial carcinomas (UC). Non-muscle invasive UC (NMIUC) accounts for 75% of BC patients and is commonly treated by transurethral tumor resection (TUR) with and without adjuvant intravesical instillations [2]. In contrast, muscle-invasive UC (MIUC) is treated with either cisplatin-based neoadjuvant chemotherapy (NAC) followed by surgical removal of the bladder (cystectomy) or external beam radiotherapy with or without chemotherapy. Despite extensive treatment, half of MIUC patients will progress to metastatic urothelial carcinoma (mUC) [3]. First-line treatment option in mUC is gemcitabine + cisplatin (“gem/cis”) [3,4]. However, 30% of mUC patients are cisplatin-ineligible due to poor performance status and other comorbidities [5,6]. Cisplatin-ineligible patients, mostly due to renal compromise, are treated with gemcitabine + carboplatin (“gem/carbo”), which is less effective than cisplatin combinations [7]. Regardless of the platinum-based chemotherapy used, most mUC patients will ultimately progress [8]. In recent years, immune checkpoint therapy (ICT) has emerged as a new option for platinum-relapsed or cisplatin-ineligible patients [3]. ICT targets cytotoxic T lymphocyte antigen 4 (CTLA4) and programmed death (ligand) 1 (PD-1/PDL1), used by tumor cells to inhibit anticancer immune responses [9]. ICT has shown superior efficacy over 2nd line chemotherapy in platinum-relapsed mUC patients [10,11,12]. Currently, several PD-1/PD-L1 inhibitors have been FDA, and EMA approved for the treatment of mUC in the first line (no prior platinum-based chemotherapy) and/or second-line (after the failure of platinum-based chemotherapy) [10,11,12,13,14]. Approved agents used PD-L1 inhibitors: atezolizumab, durvalumab, and avelumab, and PD-1 inhibitors: nivolumab and pembrolizumab [10,11,12]. Treatment of platinum-relapsed mUC patients with pembrolizumab, nivolumab, or atezolizumab was associated with an ORR of ~20% [10,11,12]. Robust biomarkers that can predict clinical response to ICT are lacking due to the complexity of tumor-immune interactions that contribute to ICT resistance [15]. However, it was found recently that a mechanism associated with resistance to ICT is metabolic competition between immune cells and cancer cells in the tumor microenvironment (TME) [16,17]. Here, we review how the metabolic profile of normal urothelial cells is reprogrammed with malignant transformation and progression and how this contributes to immune evasion and resistance to ICT. We then examine how these findings can be exploited therapeutically to enhance ICT in UC patients.

## 2. Glucose Metabolism in Urothelial Carcinoma

**Figure 1:**
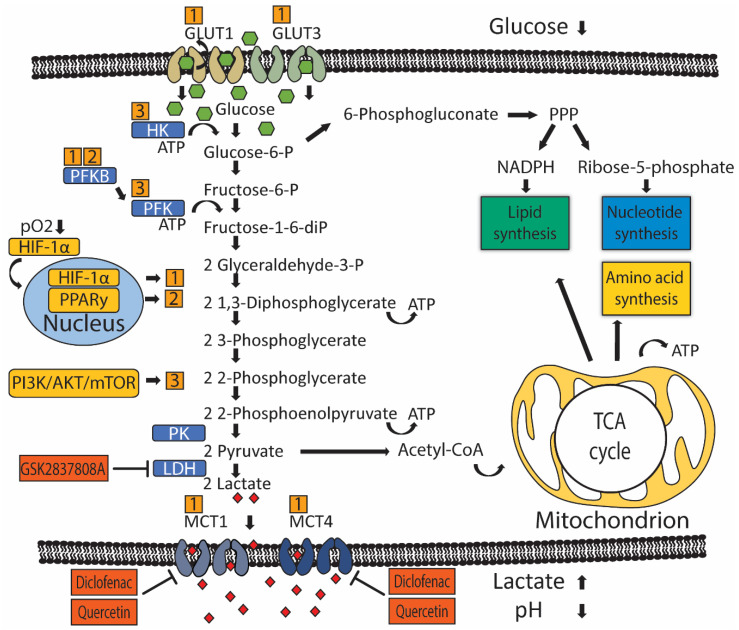
Metabolic reprogramming of glucose metabolism in UC. Oxygen-dependent hypoxia-inducible factor 1 alpha (HIF-1α) and oxygen-independent PI3K/AKT/mTOR and PPARy signaling (yellow boxes) drive metabolic reprogramming in UC by controlling the activity of rate-limiting enzymes (blue boxes) or transporters. Numbers (orange boxes) indicate which gene drives activation of particular rate-limiting enzymes or transporters. Glucose is imported into the cell by glucose transporters (GLUT) 1 and 3. Glycolysis starts with phosphorylation of glucose by hexokinase (HK) to glucose-6-phosphate, preventing glucose from diffusing outside the cell. Glucose-6-phosphate can be dehydrogenated by glucose-6-phosphate dehydrogenase (G6PD) to enter the pentose phosphate cycle (PPP) to produce pentoses like ribose-5-phosphate required for the synthesis of nucleotides and NADPH, which is necessary for reductive processes such as lipid biosynthesis. If glucose-6-phosphate is not oxidized by G6PD, glucose-6-phosphate is isomerized by phosphoglucose isomerase/phosphoglucoisomerase (PGI) to fructose-6-phosphate. Next, phosphofructokinase (PFK) catalyzes the phosphorylation of fructose-6-phosphate to fructose-1,6-biphosphate, which irreversibly channels the glucose-derived metabolite into the glycolytic pathway towards phosphoenolpyruvate. At the end of the glycolytic pathway, pyruvate kinase (PK) catalyzes the dephosphorylation of phosphoenolpyruvate (PEP) to produce one pyruvate and one ATP molecule. Pyruvate is then metabolized into lactate-by-lactate dehydrogenase (LDH). Lactate is transported outside the cell by monocarboxylate transporters (MCT) 1 and 2, which leads to an increased concentration of extracellular lactate and an acidified tumor micro-environment. Pyruvate can also be metabolized into acetyl coenzyme A (acetyl-CoA), which participates in the tricarboxylic acid (TCA) cycle inside mitochondria to give rise to ATP and intermediary metabolites that are required for lipid and amino acid biogenesis.

Glucose metabolism produces energy in the form of ATP and precursor metabolites used for biosynthesis. Glucose metabolism starts with a process called glycolysis that consists of stepwise conversions of glucose that ultimately generates pyruvate. The rate of glycolysis is regulated by hexokinase (HK), glucose-6-phosphate dehydrogenase (G6PD), phosphofructokinase (PFK), and pyruvate kinase (PK) (Figure 1). Pyruvate participates in the tricarboxylic acid (TCA) cycle, also known as the citric acid cycle (CAC), or Krebs cycle, where it is ultimately oxidized into water and carbon dioxide. This oxygen-dependent process is called oxidative phosphorylation and ultimately produces 32–38 ATP molecules from one glucose molecule.

In the absence of oxygen, pyruvate is transformed into lactate, producing 2 ATP molecules for every glucose molecule. In cancer cells, metabolic reprogramming (MR) refers to an upregulation of biosynthetic and bioenergetic pathways to produce the necessary materials and energy required for tumor growth. MR includes a shift in glucose metabolism from oxidation to glycolysis despite the presence of oxygen. This is commonly known as aerobic glycolysis [18] or the Warburg effect and favors the usage of glucose’s carbon atoms for gaining biomass (i.e., metabolic building blocks) over energy (i.e., ATP) production. Because cancer cells also need more energy, yet this arrangement is energy inefficient, there is a compensatory increase in glucose consumption [19]. Glucose uptake is seen in UC by way of positron emission tomography/computed tomography (PET/CT), which uses radioactively labeled glucose-analog fluorodeoxyglucose (^18^F-FDG) to visualize primary tumors and metastases [3]. Human UC cell lines also show increased uptake of glucose compared to untransformed urothelial cells and produce increased levels of pyruvate and lactate [20]. Evaluation of the patient’s UC tumor samples indicates glucose quantity was significantly lower compared to normal urothelium [21]. Furthermore, late TCA cycle intermediates were also increased in UC, suggesting flux into the TCA cycle to replenish intermediates extracted from the cycle for biosynthesis—a process called anaplerosis [21]. A significant increase of ribose, the end-product of the pentose phosphate pathway (PPP), was also observed in UC, suggesting upregulation of the PPP [21]. The PPP occurs in the cytosol and consists of an oxidative phase that produces NADPH, which is required for reductive processes such as fatty acid synthesis and scavenging of reactive oxygen species, and a non-oxidative phase that produces pentoses like ribose, which are important precursors for nucleotide synthesis. Therefore, the PPP helps metabolically active or proliferating cells to meet their anabolic demands and combat oxidative stress [22,23]. Thus, UC alters its metabolism and consumes glucose to produce energy via glycolysis, biomass through PPP and anaplerosis, and to counter oxidative stress through PPP.

## 3. Regulation of Glucose Transport and Metabolism in UC

Cellular glucose utilization is regulated by oxygen-dependent and oxygen-independent mechanisms that rely on several common glucose transporters and glycolytic enzymes (Figure 1). Oxygen-dependent mechanisms are mediated by transcription factor hypoxia-inducible factor 1-alpha (HIF-1α) [24,25]. Low oxygen tension stabilizes HIF-1α protein expression, which translocates to the nucleus and binds to target genes, thereby upregulating gene expression [26,27]. HIF-1α indirectly stimulates glycolysis through inhibition of mitochondrial biogenesis and oxygen consumption through induction of pyruvate dehydrogenase kinase 1 (PDK1), which subsequently inhibits pyruvate dehydrogenase from catalyzing oxidative decarboxylation of pyruvate [28,29]. The steroid receptor coactivator-3 (SRC-3) is a HIF-1α co-activator required for the expression of several HIF1-1α target genes in T24 UC cells under hypoxia [30]. Another HIF-1α co-activator is histone demethylase JMJD1A, whose H3K9 demethylase activity is required at promotor sites to induce expression of several key glycolytic enzymes [31]. Interestingly, JMJD1A was found upregulated in 46 UC patient samples, compared to 14 normal bladder samples [31]. In summary, UC has higher levels of HIF-1α co-activators, which leads to more glycolysis and reduced oxidative phosphorylation.

Oxygen-independent mechanisms of glucose utilization in UC are primarily mediated through activation of the PI3K/AKT/mTOR pathway [32,33]. The PI3K/AKT/mTOR pathway consists of activators: phosphatidylinositol 3-kinase (PI3K), protein kinase B (AKT), mammalian target of rapamycin (mTOR), and PI3K-inhibitor: phosphatase and tensin homolog (PTEN). Mutations in genes of the PI3K/AKT/mTOR pathway are present in 42/131 (38%) patients with MIUC [34]. Besides activating mutations, other factors may also promote PI3K/AKT/MTOR signaling in UC. For example, microRNA 21 (Mir-21) activates PI3K/AKT/mTOR signaling through inhibition of PTEN expression in UC cell line T24, thereby stimulating glycolysis [32,35]. Furthermore, long non-coding RNA UCA1 is associated with mTOR-mediated glucose consumption and lactate production in 5637 human bladder carcinoma cells, although no direct interaction between mTOR and UCA1 was demonstrated [33].

Peroxisome proliferator-activated receptor gamma (PPARy) has been implicated as a driver of oxygen-independent activation of glycolysis in breast cancer and hepatocellular carcinoma murine models through transcriptional activation of key glycolytic enzymes [36,37]. In UC, the increased transcriptional activity of PPARy was associated with increased mRNA expression of glycolytic enzymes and decreased recurrence-free survival in a subset of 140 non-invasive (pTa) bladder tumors [38]. Interestingly, PPARy, like PI3K/AKT/mTOR signaling, is commonly associated with the luminal subtype of MIUC, which has a relatively good prognosis [39,40,41].

Increased glycolytic flux is associated with increased glucose uptake by glucose transporters. Glucose transporter 1 (GLUT1) is the primary glucose transporter overexpressed in cancer [42]. Expression of GLUT3 has also been demonstrated in T24 UC cells [43,44]. GLUT1 protein overexpression was associated with worse overall and disease-free survival in a pooled analysis of 4079 patients with various tumor types, not including UC [45]. GLUT1 expression evaluated by immunohistochemistry (IHC) in 105 BC samples was associated with an increased grade in both NMIUC and MIUC [46]. Furthermore, GLUT1 overexpression by IHC was an independent predictor of survival following radiotherapy (N = 64) or radical cystectomy (N = 279) for MIUC [47,48]. GLUT1 expression is generally induced by HIF-1α, indicating oxygen-dependent GLUT1 expression [49]. Likewise, GLUT1 and GLUT3 expression seem also to be controlled by microRNAs that function through altering PI3K/AKT/mTOR signaling in vitro [32]. Mir-218 was found to repress GLUT1 expression and, as a consequence, glucose uptake in T24 cells, while Mir-195-5p did the same for GLUT3 [43,44]. GLUT1 knockdown elevated intracellular reactive oxygen species (ROS) and increased cisplatin sensitivity in T24 cells [43].

Once glucose is imported into the cell, hexokinase (HK) (Figure 1) is the first rate-limiting enzyme controlling glycolytic flux. HK has four isoforms characterized by different functions and cellular locations. Isoform HK2 is linked to an anabolic function through PPP and has been implicated in UC MR [33,50,51]. T24 cells overexpress HK2 in response to PI3K/AKT/mTOR signaling [32]. Pharmacological inhibition of HK2 in UC cell line UM-UC-3 lowered glucose consumption and lactate production, accentuating a potential role in UC glucose metabolism [52].

UC cells also have upregulated phosphofructokinase (PFK) (Figure 1), which drives increased glycolytic flux. Somatic genetic aberrations that upregulate or amplify PFK family genes are present in ~40% of MIUC patients [50]. In vitro studies with UC cell lines, RT4, and TCCSUP suggested that PFK is primarily important during early phases of cancer progression, as PFK expression was higher in RT4 (representing early-stage, well-differentiated NMIUC) compared to TCCSUP (representing more progressed, anaplastic MIUC) [51]. Moreover, a lower PFK activity was associated with increased pyruvate consumption, implying that more progressed tumors start to directly metabolize pyruvate instead of glucose [51]. PFK is indirectly activated by one of four 6-phosphofructo-2-kinase/fructose-2,6-biphosphatase (PFKFB) enzymes. PFKFB3 is expressed in T24, and knockdown led to decreased lactate production [53]. Another PFKFB family member, PFKFB4, was found expressed in 135 UC radical cystectomy samples [54]. High PFKFB4 expression assessed by IHC was associated with increased tumor stage and grade, and subsequent in vitro experiments demonstrated that PFKFB4 expression was induced during hypoxia in an HIF-1α-dependent manner [54].

The last step of glycolysis converts phosphoenolpyruvate (PEP) and ADP to pyruvate and ATP, and this step is catalyzed by pyruvate kinase (PK) isozymes M1/M2 (PKM1/M2) (Figure 1). UC cell lines have been shown to reexpress PKM2 [55].

In cancer cells, pyruvate is metabolized to lactate-by-lactate dehydrogenase (LDH), which reduces NADH to NAD+ in the same process. Lactate production replenishes cytosolic NAD+, allowing a continuous glycolytic flux [56,57]. Lactate produced by LDH is exported across the cell membrane by monocarboxylate transporters (MCT) in order to maintain an alkaline intracellular pH, favoring metabolism [58,59]. Tumor cells depend on MCT4 and, to a lesser extent, on MCT1 for lactate export, and MCT4 is expressed in a HIF-1α dependent manner [59,60]. MCT4 was overexpressed in approximately 50% of 360 UC patients, as assessed by IHC [61]. Moreover, MCT4 protein overexpression was an independent prognostic factor, predicting poor recurrence-free survival in NMIUC and MIUC patients treated with transurethral resection or radical cystectomy [61]. Likewise, MCT4 mRNA and protein expression predicted poor overall survival in MIUC patients treated with radical cystectomy [62]. Short interference RNA (siRNA) mediated silencing of MCT4 in UC cell lines, reduced proliferation rates, and increased ROS in a glucose-dependent manner [62]. Moreover, stable shRNA knockdown of MCT4 impaired tumor growth in an orthotopic UC xenograft model [62].

In conclusion, evidence shows that UC uses HIF-1α to increase glycolytic flux and to neutralize ROS via the upregulated activity of glucose importers (GLUT1, GLUT3), glycolytic enzymes (PFK), and lactate transporters (MCT4). Meanwhile, PI3K/AKT/mTOR signaling contributes to upregulating glycolytic enzymes (HK). Inhibiting glycolysis and lactate production may target UC either directly by impairing metabolic activity. However, most mechanistic evidence was gathered in small studies investigating parts of UC metabolism in a few human UC cell lines. More comprehensive preclinical investigation of UC metabolism in different stages of the disease is needed to increase the validity of these findings before translation into clinical trials.

## 4. Impact of UC Metabolism on Cells of the Tumor Microenvironment and Immunotherapy

**Figure 2:**
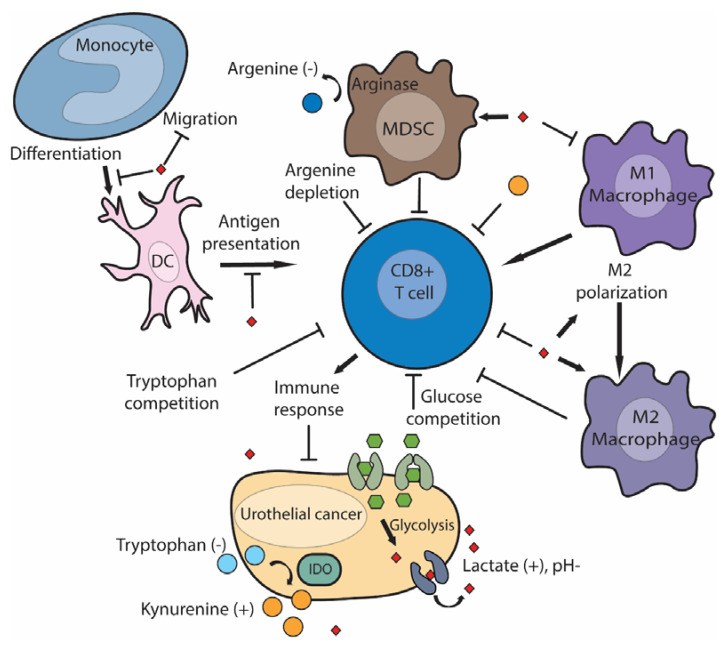
How urothelial cancer metabolism impacts immune cells in tumor microenvironment (TME)**.** Urothelial cancer creates an acidic TME deprived of glucose, tryptophan, and arginine, whereas concentrations of immune-suppressive molecules, such as lactate and kynurenine, are increased. Excessive lactate in the TME inhibits the tumor immune response by (1) polarization of macrophages to a suppressive M2 phenotype, (2) inhibition of monocyte migration and differentiation into dendritic cells (DC), thereby inhibiting antigen presentation and subsequent T cell activation, (3) by stimulating immune-suppressive myeloid-derived progenitor cells (MDSCs) to breakdown immune-stimulatory arginine, or (4) through direct inhibition of CD8+ T cell function by lactate. The T-cell-mediated immune response is also directly inhibited by tumor-produced kynurenine and indirectly through tumor-induced tryptophan and glucose shortage.

The tumor microenvironment (TME) is a critical determinant of tumor behavior and treatment response [63]. The TME is metabolically influenced by tumor cells in general, and also, UC influences the TME through MR [18,64,65]. Molecular aberrations driving MR can differ between tumor types (i.e., PI3K-activating mutations and increased PPARy-activity are characteristic drivers of UC); the outcome of MR on the TME seems to be more general and is summarized in Figure 2. Increased metabolic activity of tumor cells causes glucose and amino acid deprivation in the TME. Low glucose availability leads to metabolic competition between effector T cells and tumor cells [18,66,67], altering tumor-infiltrating lymphocytes effector function and immune response to the tumor [66,68,69]. This is in part due to T cell metabolism being glycolysis dependent and driven by HIF-1α, PI3K/AKT/mTOR, and PPARy signaling [17,64,65,70,71]. However, a recent study found that T cell viability, activation, and effector functions were preserved in a low glucose environment in vitro [66]. This suggests that restricting glucose consumption does not necessarily render T cells ineffective, possibly because T cells resort to alternative metabolic substrates. For instance, CD8+ T cells have been shown to metabolize inosine in order to produce glycolytic intermediates [67]. In line with previous observations, an artificial increase of the late glycolytic intermediate, PEP, rescued loss of T cell effector functions caused by glucose-deprivation [68]. Another study found evidence supporting the hypothesis that CD8+ T cells resort to fatty acid and self-produced ketone bodies as alternative metabolic substrates when confronted with hypoxia and glucose restrictions [69]. Interestingly, fatty acid catabolism could be further stimulated in CD8+ tumor-infiltrating lymphocytes using the selective PPARα agonist, fenofibrate [69]. Additionally, fenofibrate pretreatment of CD8+ T cells was able to slow down tumor growth in vivo, using an adoptive T cell transfer (ACT) [71]. As mentioned earlier, luminal subtype UC is driven by PPARy activation [39,41]. Therefore, a therapeutic agent that combines PPARy-antagonism with PPARα-agonism may be a suitable strategy to boost immunotherapy in bladder cancer. This evidence leads to the conclusion that glucose deprivation inhibits glycolytic activity in CD8+ T cells, but there may be ways to prevent negative consequences on T cell effector function.

Indoleamine-2,3-dioxygenase (IDO) is a rate-limiting enzyme for tryptophan metabolism, and its overexpression in cancer cells leads to tryptophan depletion in the TME, in which immune-suppressive kynurenine accumulates [72,73]. Subsequent tryptophan shortages induce a proliferation arrest in CD8+ T cells [74]. Several clinical trials have been investigating whether restoring TME tryptophan levels and preventing kynurenine accumulation through IDO-inhibition can boost ICT in UC. A phase I/IIa trial investigated IDO-inhibitor BMS-986205 in combination with nivolumab in patients with advanced UC (NCT02658890). An overall response rate (ORR) of 37% was observed among 27 ICT-naive advanced UC patients, supporting further evaluation of IDO inhibition to boost immunotherapy [75]. However, it is difficult to interpret the high ORR of 37% without a comparison arm receiving only nivolumab treatment or information on PD-L1 expression. Another phase I study is currently investigating the safety profile of IDO-inhibitor KHK2455 in combination with avelumab in platinum-relapsed metastatic UC patients (NCT03915405). The PECULIAR phase II trial is investigating neoadjuvant pembrolizumab combined with IDO-inhibitor epacadostat in MIUC patients prior to radical cystectomy (NCT03832673). Epacadostat will also be evaluated as a neoadjuvant treatment option in a phase II umbrella study that allocates treatment according to FGFR3 mutation status (NCT04586244). Treatment with either epacadostat alone, or in combination with anti-PD-1 monoclonal antibody retifanlimab will be available for MIUC patients without FGFR3 mutations or fusions, who do not qualify for FGFR-directed therapy (NCT04586244). Two phase III studies are currently investigating clinical efficacy of pembrolizumab + epacadostat in platinum-relapsed mUC patients (NCT03374488, NCT03361865). Moreover, safety and preliminary efficacy of IDO1 inhibitor epacadostat in combination with arginase inhibitor INCB001158 and pembrolizumab is under investigation in patients with solid tumors, including mUC (NCT03361228).

It has been demonstrated that oxygen-tension in the TME could be raised to benefit immunotherapy by inhibiting oxidative phosphorylation in vivo [76]. Metformin is a drug widely used for diabetic patients that uncouples oxidative phosphorylation. Metformin monotherapy was found to decrease tumor growth of 5637 human bladder carcinoma cells in nude mice [77] and boost the efficacy of anti-PD-1 therapy (J43) in a murine melanoma model [76]. A phase 2 clinical trial is investigating the preliminary efficacy of metformin in combination with nivolumab for treating metastatic lung cancer patients (NCT03048500).

A hypoxic TME drives oxygen-dependent upregulation of UC’s glucose consumption and lactate production. Lactate is exported extracellularly, where concentrations can rise up to 40 mM compared to the physiological concentration of 1–3 mM [78,79,80,81]. The high lactic acid in the TME promotes angiogenesis, metastasis, and immune escape [82].

Tumor-immune responses are initiated by tumor-associated dendritic cells. Tumor-associated dendritic cells develop from monocytes and play a central role in tumor immunity by capturing neoantigens and presenting neoantigens to T cells, which initiates a T-cell-mediated immune response directed at neoantigen expressing tumor cells [83]. Lactate concentrations of 20nM inhibited both monocyte migration and the release of immune-stimulatory cytokines like interleukin-6 and tumor necrosis factor (TNF) [84]. In addition, lactic acid changes the differentiation of monocytes into dendritic cells and inhibited antigen presentation of dendritic cells [85]. Tumoral lactate production was also found to increase the presence of myeloid-derived suppressor cells (MDSC), which have inhibitory effects on cytotoxic T cells [86]. Recently, it was discovered that MDSC suppress T cells via the transfer of the glycolytic byproduct methylglyoxal, which had an inhibitory effect by depleting L-arginine in CD8+ T cells, thereby paralyzing antitumor immunity [87]. Two clinical trials are currently investigating the possibility of restoring arginine levels to enhance immunotherapy. A phase 1 trial enrolling up to 260 patients with metastatic solid tumors, including UC patients, is investigating arginase inhibitor INCB001158 in combination with pembrolizumab (NCT02903914). Arginase is produced by MDSC and breaks down proinflammatory arginine thus blocking arginase activity and may partially restore antitumor immune responses. Likewise, the safety of a cell-permeable l-arginine analog, NG-monomethyl-L-arginine (L-NMMA), is being investigated in combination with pembrolizumab in a phase 1b trial also enrolling UC patients (NCT03236935).

Tumor-associated macrophages (TAMs) are generally considered unfavorable, driving tumor progression and metastasis [88]. However, TAMs have a dual role, as pro-inflammatory M1 macrophages with anti-tumor activity and M2 macrophages promoting tumor growth and immune escape [89]. Lactate produced by tumor cells has been shown to polarize M1 macrophages to an M2 phenotype mediated through increased expression of HIF-1α in TAMs [90,91]. This was first demonstrated in vitro when TAMs from patients showed increased HIF-1α expression and M2 polarization when treated with either lactate or conditioned medium derived from Lewis lung carcinoma (LLC) and melanoma cell lines in vitro [90]. A similar mechanism has been shown for bladder cancer, as either co-culture of murine RAW 264.7 macrophages with T24 UC cells or lactate treatment inhibited M1 polarization, while M2 polarization was induced [91]. It remains to be determined how lactate exactly increases M2 polarization in TAMs, but the mechanism seems to depend on HIF-1α expression and the intracellular lactate levels of macrophages, as the effects of lactate treatment on TAM polarization could be averted through the blocking of lactate transporters with MCT-inhibitor quercetin or HIF-1α knockdown [90,91]. Thus, extracellular lactate can be immune suppressive by decreasing the presence and activity of tumor-associated dendritic cells while activating MDSC-mediated immune suppression and driving M2 macrophage polarization.

Lactate production can also decrease T-cell function [92,93,94]. The high presence of lactic acid in the TME reduces the gradient-dependent export of lactate through MCT1, thereby disturbing cytotoxic T cell metabolism as intracellular lactate accumulates and intracellular pH drops [93,94]. Interestingly, extracellular sodium lactate (associated with neutral/increased pH) was found to inhibit CD4+ T helper cell motility and stimulate the production of proinflammatory interleukin-17, whereas lactic acid (associated with decreased pH) caused the loss of cytolytic activity of CD8+ T cells [92]. Whereas, an extracellular lactate concentration of 20 mM, measured by the routinely used AVDIA 1650 chemical analyzer, was enough to decrease proliferation and cytokine production of cytotoxic T cells by 95%, while cytotoxic activity towards melanoma cells was decreased by 50% [94]. Moreover, intratumoral pH measured by microelectrode could be increased from pH 6.8 to pH 7.0 in melanoma xenografts fed with 200 mM bicarbonate drinking water [95]. Neutralizing tumor acidity with oral bicarbonate treatment synergized with either adoptive T cell transfer, anti-CTLA4 or anti-PD-1 treatment in two melanoma xenograft models [95]. Safety of bicarbonate supplementation in humans has been assessed in 15 healthy volunteers, and dosage of up to 0.17 g/kg/day for 90 days was well tolerated (0.5 g/kg/day was the predicted equivalent of 200 mM bicarbonate drinking water fed to the xenografts), suggesting that moderate, oral bicarbonate supplementation might be feasible for cancer patients [96]. These results imply that the earlier described balance between immune-stimulatory sodium lactate and immune-suppressing lactic acid could be used as a switch to turn on the tumor immune response, although no clinical trials have been reported to investigate bicarbonate treatment with immunotherapy.

LDH knockdown in melanoma xenograft model diminished intratumoral lactate concentrations while increasing concurrent CD8+ T cell tumor infiltration and reducing tumor growth in a melanoma xenograft model [93]. Most recently, shRNA knockdown of LDH synergized with anti-PD-1 treatment while confirming that limiting lactate production led to increased infiltration of T cells and NK cells [97]. Nonetheless, treatment with LDH-inhibitor GSK2837808A was additive but not synergistic with adoptive T cell transfer treatment in another melanoma xenograft model [98]. That pharmacological inhibition of LDH is additive to immunotherapy, but that genetic inhibition is synergistic may be explained by metabolic similarities between immune and cancer cells [17]. In contrast to the described lack of synergism when lactate production is targeted using a pharmacological approach, there has been reported synergism with diclofenac-mediated inhibition of lactate transporters MCT1 and MCT4, present both on tumor cells and T cells, in combination with anti-PD1 and anti-CTLA4 therapy in melanoma xenografts [68]. This indicates that blocking lactate transport may be a window of opportunity to boost immunotherapy. Clinical evidence for the hypothesis that a high glycolytic flux and increased lactate production hampers immunotherapy was obtained in 47 melanoma patients who received anti-PD-1 therapy [68]. Patients with a predicted high glycolytic index, based on mRNA expression of glycolytic enzymes, had a worse progression-free survival than patients with tumors that had a low glycolytic activity [68]. At present, there are no reported clinical trials investigating the effects of targeted glucose metabolism or lactate production in combination with ICT (Table 1).

## 5. Concluding Remarks and Future Perspectives

The TME is a complex environment that includes cells with both proinflammatory as well as immune suppressive responses. Metabolic reprogramming of urothelial cells that undergo transformation into UC is exemplified by hypoxia, PPARy and/or PI3K/mTOR/AKT signaling-mediated upregulation of PPP, increased anaplerosis through the TCA, and most importantly, increased glycolysis and lactate production.

These changes deprive the TME of nutrients, while extracellular, immune-suppressive molecules like lactic acid or kynurenine increase, thereby contributing to immune evasion. Combining ICT with approaches that target UC metabolism may benefit the metabolic demands of immune cells with antitumor activity such as M1 macrophages or CD8+ T cells. Clinical trials are currently combining ICT with drugs targeting tumor-induced arginine-depletion or immune-suppressive metabolites in UC patients. Arginase depletion can be countered through arginase-inhibition, which is being evaluated in combination with pembrolizumab (NCT02903914). Additionally, IDO-inhibition could overcome both tryptophan depletion and accumulation of immune-suppressive kynurenine. IDO-inhibition is under investigation in combination with pembrolizumab (NCT03374488, NCT03361865), nivolumab (NCT02658890), avelumab (NCT03915405), and retifanlimab (NCT04586244). Strategies that inhibit UC glycolysis and lactic acid production seem promising methods to boost the effectiveness of ICT, although further exploration for cancer-specific and localized targeting is warranted in order to protect noncancerous cells from glucose deprivation. Preclinical models have demonstrated that disrupting lactate transport with diclofenac-mediated MCT-inhibition, neutralizing intratumoral pH with oral bicarbonate treatment, or counteracting intratumoral hypoxia with metformin can synergize with ICT [68,76]

In conclusion, disrupting UC metabolism may be a new approach to boost ICT for BC patients. Novel druggable targets are being explored in preclinical models, but the repurposing of existing drugs to disrupt the glucose metabolism (metformin) or lactate transport (diclofenac) and boost ICT should be investigated as well.

## Figures and Tables

**Table 1:** Clinical trials investigating metabolic reprogramming for immunotherapy.

Drug	Target	Combination Therapy	Setting	Identifier
INCB001158	Arginase	Pembrolizumab	Solid tumors, including UC	NCT02903914
L-NMMA	Nitric oxide synthase	Pembrolizumab	Solid tumors, including UC	NCT03236935
BMS-986205	IDO	Nivolumab	mUC	NCT02658890
KHK2455	IDO	Avelumab	Platinum-relapsed mUC	NCT03915405
Epacadostat	IDO	Pembrolizumab	Platinum-relapsed mUC	NCT03374488, NCT03361865
Epacadostat	IDO	Pembrolizumab	Neoadjuvant MIUC	NCT03832673,
Epacadostat	IDO	Retifanlimab	Neoadjuvant MIUC	NCT04586244
Epacadostat, INCB001158	Arginase, IDO	Pembrolizumab	Solid tumors, including UC	NCT03361228
Metformin	Hypoxia, oxidative phosphorylation	Nivolumab	NSCLC	NCT03048500

IDO = indoleamine 2,3-dioxygenase, UC = urothelial cancer, mUC = metastatic urothelial cancer, MIUC = muscle-invasive bladder cancer, NSCLC = non-small cell lung cancer.
